# Dynamic immune landscape of NAFLD highlights pDCs as predictive biomarkers for disease progression

**DOI:** 10.3389/fimmu.2026.1702007

**Published:** 2026-04-10

**Authors:** Guozhen Huang, Siyi Xing, Zhaojun Long, Wei Gao, Shaolei Kuang, Mingjiang Liu, Yan Zou, Chenyi Zhuo, Xiaofeng Dong

**Affiliations:** 1 Department of Hepatobiliary, Pancreas and Spleen Surgery, The People’s Hospital of Guangxi Zhuang Autonomous Region (Guangxi Academy of Medical Sciences), Nanning, China; 2 Guangxi Key Laboratory of Immunology and Metabolism for Liver Diseases, Guangxi Medical University, Nanning, China; 3 School of Life Science, Hubei University, Wuhan, Hubei, China; 4 Ultrasound Medicine Department, Jiangbin Hospital of Guangxi Zhuang Autonomous Region, Nanning, China; 5 Key Laboratory of Molecular Pathology for Hepatobiliary Diseases of Guangxi, Affiliated Hospital of Youjiang Medical University for Nationalities, Baise, China

**Keywords:** diagnostic markers, immune landscape, machine learning, NAFLD, pDCs

## Abstract

**Introduction:**

Non-alcoholic fatty liver disease (NAFLD) is associated with profound alterations in the hepatic immune microenvironment; however, a comprehensive understanding of immune cell dynamics across disease stages remains limited.

**Methods:**

We performed an integrative reanalysis of publicly available single-cell transcriptomic datasets from the GEO database, encompassing human liver samples spanning the NAFLD continuum. Candidate immune signatures were validated in independent human cohorts and experimentally induced murine NASH models. A diagnostic classifier leveraging pDC associated gene profiles was developed and rigorously evaluated across multiple validation cohorts.

**Results:**

Analysis revealed extensive stage-dependent remodeling of hepatic immune composition, with pDCs exhibiting the most pronounced transcriptional and numerical shifts. Specifically, a unique pDC subset coexpressing GZMB and TPM2 was significantly enriched in human NASH samples. This signature was consistently recapitulated in independent human datasets and murine NASH models. The pDC-derived diagnostic model demonstrated robust performance for NASH identification across all validation cohorts.

**Conclusion:**

GZMB^+^TPM2^+^ pDCs constitute a conserved, NASH-specific immune signature validated across species. The associated diagnostic model provides a robust, clinically applicable tool for non-invasive NASH identification.

## Introduction

Non-alcoholic fatty liver disease (NAFLD), also termed metabolic dysfunction-associated steatotic liver disease (MASLD), represents the most prevalent global liver condition, spanning a spectrum from simple steatosis to non-alcoholic steatohepatitis (NASH), cirrhosis, and hepatocellular carcinoma (1, 2). Despite advancements in hepatitis C and B management, NAFLD remains the leading cause of liver-related morbidity and transplantation in developed nations (3). However, disease progression is highly heterogeneous: only ~20% of simple steatosis cases progress to NASH, with ~30% subsequently developing cirrhosis (4, 5). The molecular mechanisms driving this variability remain poorly defined.

Immune dysregulation involving liver-resident dendritic cells (DCs) is central to NASH pathogenesis. Among DC subsets, plasmacytoid DCs (pDCs) are of particular interest as primary type I interferon producers. Oxidized mitochondrial DNA selectively activates pDCs to secrete IL-1β, promoting pro-inflammatory T-cell differentiation (6), and hepatic pDC infiltration strongly correlates with fibrosis severity in human MASLD (7). These observations position pDCs as a critical nexus between metabolic stress and immune activation. However, the regulatory mechanisms governing pDC functionality in NAFLD, especially immune checkpoint pathways, remain inadequately characterized.

A key regulatory axis involves the antagonistic signaling of B and T lymphocyte attenuator (BTLA) and activated leukocyte cell adhesion molecule (ALCAM). BTLA functions as a co-inhibitory receptor that engages herpes virus entry mediator (HVEM) (8), suppressing DC over-maturation and pro-inflammatory cytokine release while directly restraining T-cell activation at the immunological synapse (9). This pathway serves as a molecular brake for hepatic immune tolerance. In contrast, ALCAM is upregulated during DC maturation and mediates transendothelial migration to inflammatory sites while stabilizing DC–T-cell interactions to ensure efficient antigen presentation (10), thereby acting as a molecular accelerator of immune responses. Although these pathways are well established in systemic immunity and other inflammatory diseases, their specific roles in modulating pDC behavior during NAFLD progression have not been elucidated, representing a significant knowledge gap with direct relevance to disease heterogeneity.

To address this gap, we leveraged single-cell RNA sequencing (scRNA-seq) to perform an integrated meta-analysis of public datasets spanning healthy controls, steatosis, NASH, and cirrhosis. We identified a progressive expansion and transcriptional reprogramming of hepatic pDCs specifically in NASH. Building on this signature, we developed and validated a minimal five-gene pDC-derived classifier with robust diagnostic performance for distinguishing NASH from non-NASH conditions. These findings not only illuminate pDCs as dynamic contributors to MASLD progression but also establish a clinically translatable framework for precision staging.

## Materials and methods

### Single-cell RNA-sequencing data collection and quality control

Single-cell transcriptomic data were collected from three public datasets (GSE115469, GSE159977, and GSE136103) comprising 30 scRNA-seq samples from 22 human liver donors, stratified into four groups based on liver biopsy pathology: healthy (14 samples), borderline NASH (bNASH; three samples), NASH (eight samples), and cirrhosis (five samples) (11–13). Specifically, GSE115469 included five healthy donors with whole-liver dissociation (no cell selection), yielding approximately 10,000 cells. GSE159977 encompassed four healthy, three bNASH, and three NASH donors, where Fluorescence Activated Cell Sorter (FACS)-sorted immune cells (CD45^+^ enrichment) were profiled, generating ~50,000 cells. Meanwhile, GSE136103 comprised five healthy and two cirrhosis donors with CD45^+^ selection, separating cells into CD45^+^ (immune) and CD45^−^ (non-immune) fractions, contributing ~98,000 cells in total. The bNASH group represents transitional biopsy samples exhibiting histopathological features intermediate between non-alcoholic fatty liver (NAFL) and definitive NASH, characterized by mild lobular inflammation and/or borderline ballooning degeneration without advanced fibrosis. The NASH and cirrhosis groups were defined by clear pathological hallmarks: NASH samples showed ≥2 of the following features—steatosis, lobular inflammation, and hepatocyte ballooning—while cirrhosis samples exhibited extensive fibrosis with architectural distortion. A total of 158,901 cells were analyzed pre-quality control. To ensure robust cell subset annotation, cells with <200 detected genes, <200 gene features, or >20% mitochondrial gene content were excluded, retaining 156,299 high-quality cells for downstream analysis. All datasets were integrated, and batch effects were removed using Seurat’s Harmony package prior to analysis.

### Clustering and cell-type identification

The R Seurat v4−(v4.4.0) and harmony (v1.2.0) packages were applied to analyze the expression matrix (14, 15). The count matrix was normalized with default settings, and 2,000 variable features identified were scaled and used for Principal Component Analysis (PCA). Harmony-corrected PC1–PC50 were used to construct a K-nearest neighbor graph with K = 21 and to identify unsupervised cell clusters. Resolutions ranging from 0.1 to 1.0 were chosen for different cell cluster identifications using the R clustree (v0.5.1) package (16). Cluster-specific gene markers were identified using the FindAllMarkers function in Seurat with log2(fold change) > 0.25 and min.pct (minimum fraction) > 0.25. For dimension reduction, a Uniform Manifold Approximation and Projection (UMAP) was implemented to visualize cell clustering. Cell types were identified mainly based on the canonical markers for major cell types and published articles, with the ScType algorithm and the Annotation of Cell Types (ACT) website (17, 18). First, this workflow was carried out on all single cells to identify major cell types, including hepatocytes, cholangiocytes, myeloid cells, lymphocytes, mesenchymal cells, and endothelial cells. Then, the workflow was reapplied to identify variable genes across immune cells within the same major cluster and built cell embeddings for subset identification. A total of 14 subsets in 14,650 myeloid cells and 19 subsets in 119,078 lymphocytes were identified. The R plot1cell (v0.0.0.9000) package was used for the visualization of single-cell data (19).

### Differential expression and enrichment analyses

To perform differential expression analysis, Wilcoxon rank-sum tests were applied on the genes expressed in >25% of the cells in either group being compared. Genes with adjusted p < 0.05 and absolute log2(fold change) > 0.585 were considered differentially expressed genes (DEGs). For enrichment analysis, the R clusterProfiler (v4.10.1) and GSVA (v1.50.1) packages were used to identify Gene Ontology (GO) and Kyoto Encyclopedia of Genes and Genomes (KEGG) Enrichment Analysis terms, and p-values < 0.05 were considered statistically significant (20, 21). Heatmaps for biological process (BP) from each immune cell type were plotted using the R pheatmap (v1.0.12) package (22), Venn diagrams were generated using the R VennDiagram (v1.7.3) package (23), and the correlation heatmap of immune cells was plotted using the R corrplot (v0.92) package (24).

### pDC–lymphocyte interaction identification

The R CellChat (v1.6.1) package was used to infer cell–cell interactions between pDCs and lymphocytes (25). Ligand–receptor pairs with p < 0.05 were considered in the downstream analysis and visualization.

### Clustering, differential expression, and trajectory analysis for pDCs

Trajectory analysis was conducted on pDCs using the R monocle (v2.30.0) package (26). The pDCs (Healthy) were utilized to aid in determining the direction of cell differentiation. By testing each gene for differential expression with different cell states and identifying genes with branch-dependent expression, a comprehensive analysis of the differential differentiation trajectories between pDCs (NASH) and pDCs (Cirrhosis) was conducted. The R ClusterGVis (v0.1.2) package was used for the visualization of DEGs and GO enrichment (27).

### Construction, evaluation, and validation of the diagnostic model

The datasets GSE126848, GSE89632, GSE135251, and GSE167523 were downloaded from the National Center for Biotechnology Information-GEO Gene Expression Omnibus (GEO) database. The GSE126848 dataset (based on the GPL18573 platform) consisted of 16 samples from NASH patients and 14 samples from healthy individuals (28). The GSE89632 dataset (based on the GPL14951 platform) comprised 19 samples from NASH patients and 24 samples from healthy individuals (29). The GSE135251 dataset (based on the GPL18573 platform) comprised 155 samples from NASH patients (according to histopathological disease grade and stage: F1, F2, F3, and F4) (30). The GSE167523 dataset (based on the GPL21290 platform) consisted of 47 samples from NASH patients (31).

DEGs of GSE126848 and GSE89632 were identified using the R limma (v3.58.1) package (32). p-Value < 0.05 and absolute log2(fold change) > 0.585 were defined as DEGs. A Venn diagram was used to show the intersection of DEGs for GSE126848, GSE89632, and Branch Point 2 of pDCs. The diagnostic values of IFI6, FBXO8, SPINT2, UNC119, and CEBPD for NASH were determined by generating receiver operating characteristic (ROC) curves and calculating the area under the curve (AUC) values using the R pROC (v1.18.5) package. A two-sided p-value < 0.05 was considered statistically significant (33). Furthermore, the random forest (RF), support vector machine (SVM), and eXtreme Gradient Boosting (XGBoost) algorithms were used to calculate the AUC values for NASH using the R mlr3 (v0.18.0) package (34). The R forestmodel (v0.6.2) and rms (v6.8.0) packages were used for visualization for a fit generalized linear model and a partial nomogram model (35, 36). Unsupervised K-means consensus clustering was performed for 47 NASH samples (GSE167523) using the R ConsensusClusterPlus (v1.66.0) package (37). The average pairwise consensus matrix for consensus clusters, the delta plot of the relative change in the area under the cumulative distribution function curve, and the average silhouette distance within consensus clusters were compared, and k = 2 was selected as the best sample subgrouping.

### Murine model of NASH

This study was conducted in compliance with the Animal Research: Reporting of In Vivo Experiments (ARRIVE) guidelines for animal research. Male C57BL/6J mice (8–10 weeks old) were fed a methionine- and choline-deficient (MCD) diet for 8 weeks to induce NASH. Control mice received a standard chow diet under standardized conditions. Hepatic steatosis was confirmed via ultrasound imaging at the end of the feeding period. NASH phenotypes (steatosis, inflammation, and fibrosis) were rigorously validated in all subjects via histological analysis and serum Alanine Aminotransferase (ALT) & Aspartate Aminotransferase (AST). profiling prior to euthanasia. Animals were humanely euthanized by exposure to 4% isoflurane (inhaled via precision vaporizer), with death confirmed by the absence of respiration, heartbeat, and corneal reflex. Whole blood, liver tissues, and perfused organs were collected immediately post-mortem for downstream analysis. All procedures were approved by the Institutional Animal Care and Use Committee of the People’s Hospital of Guangxi Zhuang Autonomous Region (Approval No. KY-GZR-2021-043) and conducted in accordance with the American Veterinary Medical Association (AVMA) Guidelines for the Euthanasia of Animals.

### Human NAFLD samples

Liver biopsy samples were obtained from patients diagnosed with NAFLD at the People’s Hospital of Guangxi Zhuang Autonomous Region (Ethics Approval No. KY-GZR-2021-043) and the First Affiliated Hospital of Guangxi Medical University (Ethics Approval No. KY20250235). Healthy control tissues were derived from morphologically normal liver regions of individuals undergoing elective liver transplantation or hepatectomy, with no evidence of liver disease. All human studies were approved by the respective institutional ethics committees and conducted in strict compliance with the Declaration of Helsinki (2013). Written informed consent was obtained from all patients prior to sample collection, and all data were anonymized to protect participant confidentiality. The original consent forms are archived at the institutional ethics committees in accordance with national regulations.

### Histological and immunofluorescence analyses

For histological evaluation, liver tissues were fixed in 4% paraformaldehyde, embedded in paraffin, sectioned, and stained with H&E. Immunofluorescence staining was performed on deparaffinized sections after antigen retrieval. Sections were incubated with primary antibodies, followed by fluorescent secondary antibodies and 4',6-diamidino-2-phenylindole (DAPI) counterstain. Multiplex immunofluorescence labeling was performed using the ABclonal TSA Fluorescence Double Staining Kit (RK05902-50T) with primary antibodies against GZMB [Proteintech (Wuhan, Hebei), 13588-1-AP] and TPM2 [Proteintech (Wuhan, Hebei), 11038-1-AP].

## Results

### Characterization of the liver immune landscape across different stages of NAFLD

**Figure 1:**
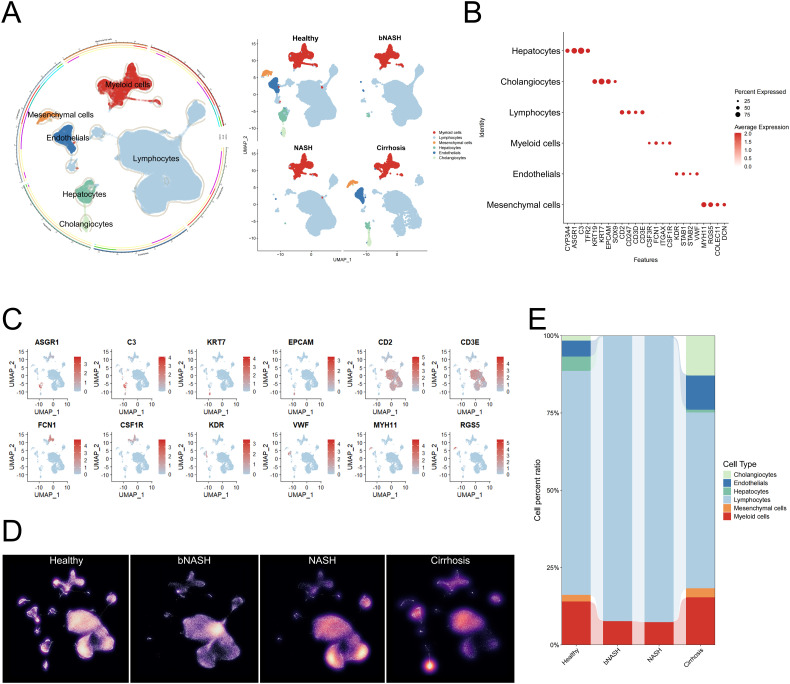
Integrated single-cell transcriptomic profiling of NAFLD. **(A)** Circular schematic plot illustrating liver cell clustering in NAFLD patients, with distinct colors representing different cell types. **(B)** Key molecular markers defining each identified cell type. **(C)** UMAP plot depicting the expression patterns of selected molecular markers within immune cell populations. **(D)** UMAP density plots showing cell distribution across disease stages (Healthy, bNASH, NASH, and Cirrhosis). High-density regions are indicated in yellow, while low-density areas are shown in black. **(E)** Stacked bar chart illustrating dynamic shifts in immunophenotypes during NAFLD progression. NAFLD, non-alcoholic fatty liver disease; UMAP, Uniform Manifold Approximation and Projection; NASH, non-alcoholic steatohepatitis; bNASH, borderline NASH.

To systematically delineate immune cell dynamics throughout NAFLD progression, we integrated single-cell transcriptomic datasets from three public repositories (GSE136103, GSE115469, and GSE159977), encompassing four distinct pathological states: healthy controls, bNASH, established NASH, and cirrhosis (**Figure 1A**). Following rigorous normalization and batch-effect correction to ensure cross-dataset comparability, unsupervised clustering based on canonical signature genes revealed stage-specific compositional shifts across hepatic cellular populations (**Figures 1A–E**).

**Figure 2:**
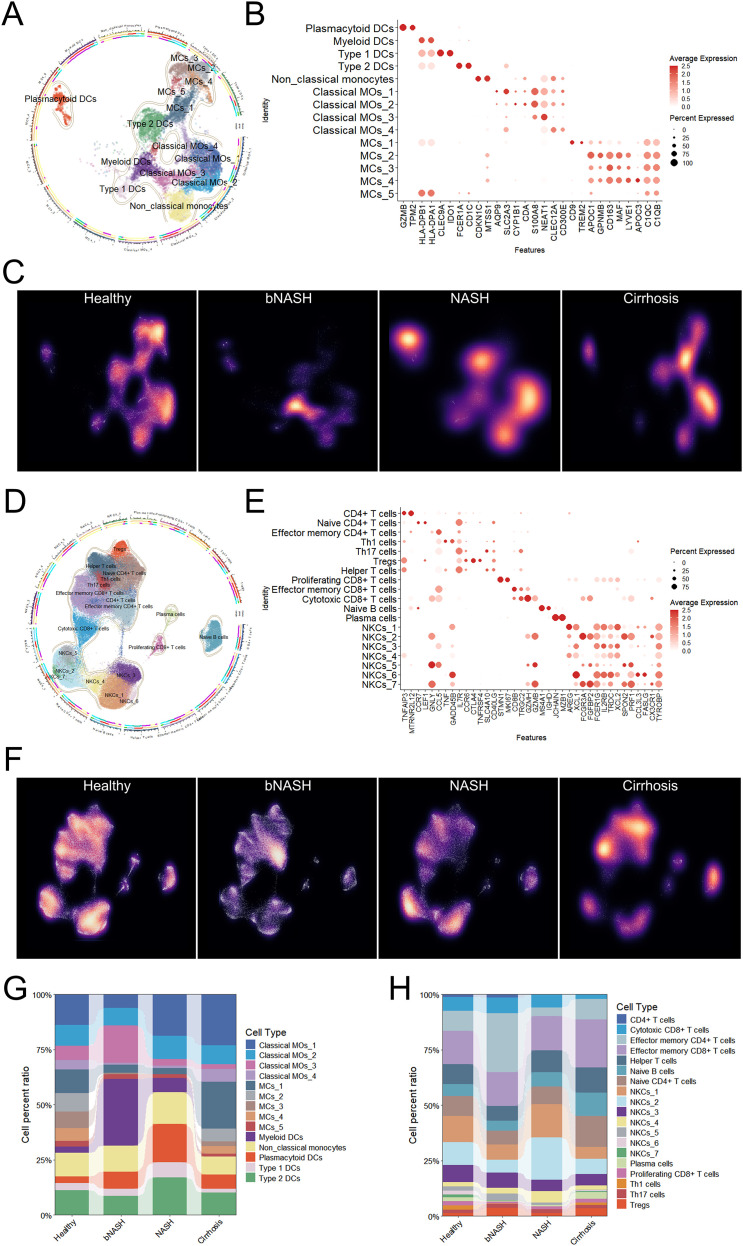
The liver immune landscape in NAFLD. **(A)** Schematic circular plot of myeloid cell subclustering. **(B)** Molecular markers specific to each myeloid cell cluster. **(C)** UMAP density plot illustrating the spatial distribution of myeloid cells in NAFLD patients. **(D)** Schematic circular plot of lymphocyte subclustering. **(E)** Molecular markers identifying distinct lymphocyte clusters. **(F)** UMAP density plot displaying the localization of lymphocyte subsets in NAFLD patients. **(G, H)** Stacked bar chart summarizing dynamic changes in immune cell composition across changes across liver pathology stages, with myeloid cells **(G)** and lymphocytes **(H)** separately displayed. NAFLD, non-alcoholic fatty liver disease; UMAP, Uniform Manifold Approximation and Projection.

Given the pivotal role of immune cells in NAFLD pathogenesis, we established a high-resolution hepatic immune atlas spanning the continuum from homeostasis to end-stage fibrosis (**Figures 2A–F**; **Supplementary Figures 1A, B**). Distinct immunological signatures emerged at each disease stage (**Figures 2G, H**). In bNASH, significant expansions were observed in myeloid dendritic cells (moDCs), classical monocyte subset 3 (cMo-3), pDCs, effector memory CD4^+^ T cells, and regulatory T cells (Tregs), indicating early inflammatory activation. Conversely, reductions in macrophages, Th1 cells, and NK cells subset 1 (NKCs-1) suggested incipient immunoregulatory imbalance (**Figures 2G, H**). Notably, certain populations exhibited minimal abundance changes yet underwent profound transcriptional reprogramming at the subset level.

Advancement to established NASH manifested further immune remodeling: diminished moDCs, effector memory CD4^+^ T cells, and Tregs coincided with expanded pDCs, cDC2s, Th, and NKCs. Intriguingly, during fibrogenesis, immune composition partially reverted toward a profile resembling homeostasis; however, this apparent normalization likely reflects adaptive immune network rewiring rather than true physiological recovery. Collectively, these findings delineate a dynamically evolving hepatic immune landscape across NAFLD progression, thereby pinpointing critical immunological checkpoints for therapeutic targeting.

### Transcriptional profiling of pDCs in NAFLD

**Figure 3:**
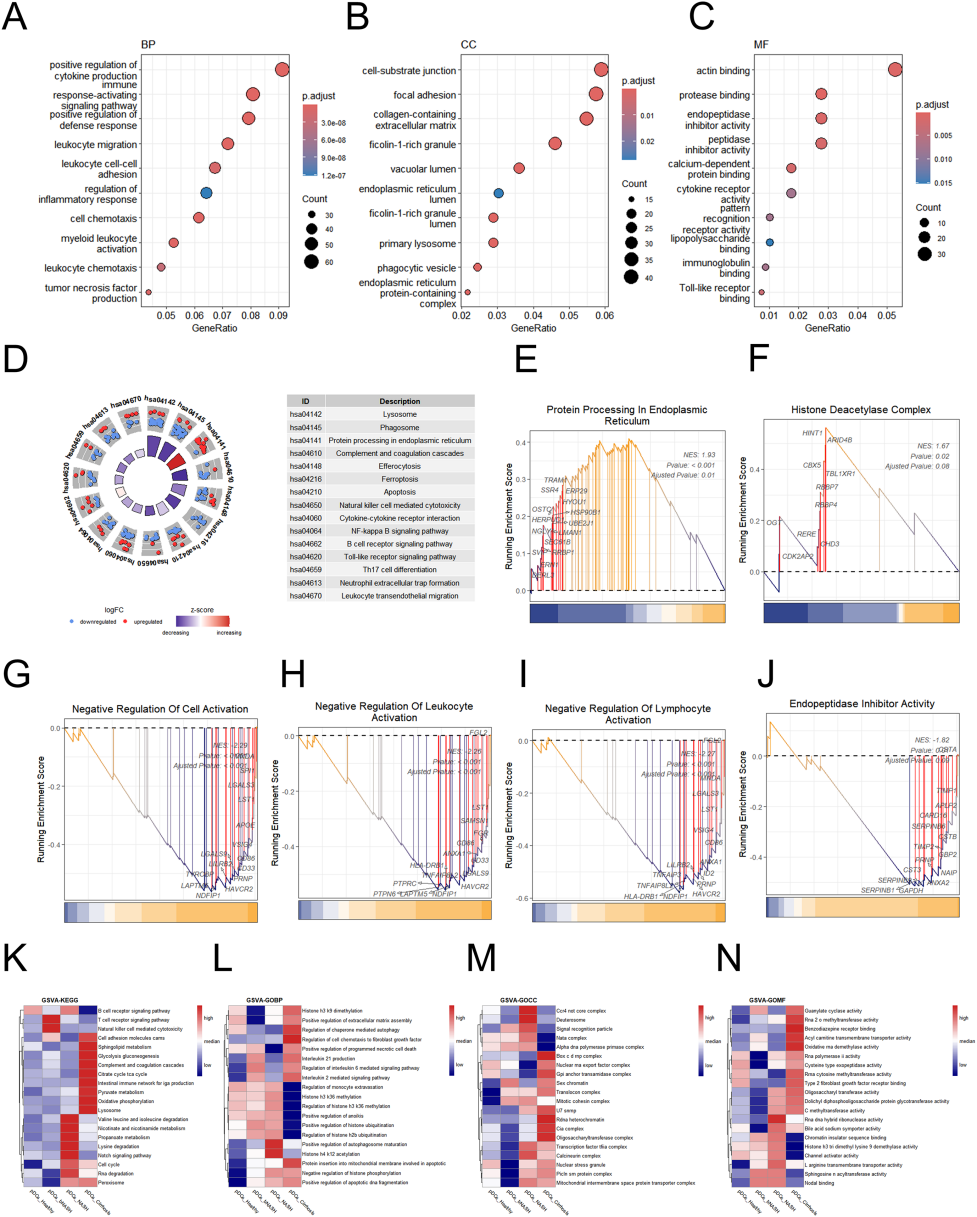
Enrichment and functional characterization of pDCs in NASH livers. **(AC)** GO enrichment analysis of pDCs, including BP **(A)**, CC **(B)**, and MF **(C)**. **(D)** Circular plot (left) presenting KEGG pathway enrichment results for pDCs, with corresponding pathway annotations listed on the right. **(E–J)** GSEA of activated **(E, F)** and suppressed **(G, J)** molecular pathways in pDCs during NAFLD progression. **(K–N)** Heatmaps show distinct GSVA enrichment profiles of pDCs across different stages of NAFLD. NAFLD, non-alcoholic fatty liver disease; pDCs, plasmacytoid dendritic cells; NASH, non-alcoholic steatohepatitis; GO, Gene Ontology; BP, biological process; KEGG, Kyoto Encyclopedia of Genes and Genomes; CC, Cellular Component; MF, Molecular Functionm; GSEA, Gene Set Enrichment Analysis; GSVA, Gene Set Variation Analysis.

pDCs constitute the most markedly expanded immune subset in NASH-stage livers, yet their functional contributions remain poorly defined. To elucidate pDC contributions to NAFLD pathogenesis, we performed comprehensive transcriptomic analysis of pDC populations and conducted GO and KEGG pathway enrichment analyses on differentially expressed genes. GO analysis revealed that pDCs are central to immune response activation and regulation of inflammatory cytokine production (**Figures 3A–C**). KEGG pathway analysis revealed that the predominant active pathways in pDCs during NAFLD were complement system signaling, efferocytosis, ferroptosis, apoptosis, and NK cell-mediated cytotoxicity (**Figure 3D**).

Comparative analysis against other myeloid populations revealed the upregulation of protein processing in the endoplasmic reticulum (ER) and histone deacetylase complex pathways, concomitant with the downregulation of the negative regulation of cell activation and leukocyte activation pathways (**Figures 3E–J**). While core functions remained consistent across stages, NASH-stage pDCs exhibited distinct molecular signatures, including valine, leucine, and isoleucine degradation, nicotinate and nicotinamide metabolism, and the Notch signaling pathway—all of which are fundamentally important for maintaining cellular energy homeostasis (**Figures 3K–N**; **Supplementary Figures 1C, D**). As essential precursors of NAD^+^ and NADP^+^, these metabolites directly influence redox reactions, mitochondrial function, and key enzymatic processes in glycolysis, β-oxidation, and the tricarboxylic acid (TCA) cycle. Dysregulation of this pathway has been increasingly recognized as a contributing factor to metabolic disorders, including NAFLD, where impaired NAD^+^ availability compromises mitochondrial efficiency and promotes hepatic lipid accumulation (38, 39). These results suggest that pDCs may contribute to the development of NASH by modulating these pathways.

### Immune regulatory mechanisms of pDCs involving Tregs and NK cells in NAFLD

**Figure 4:**
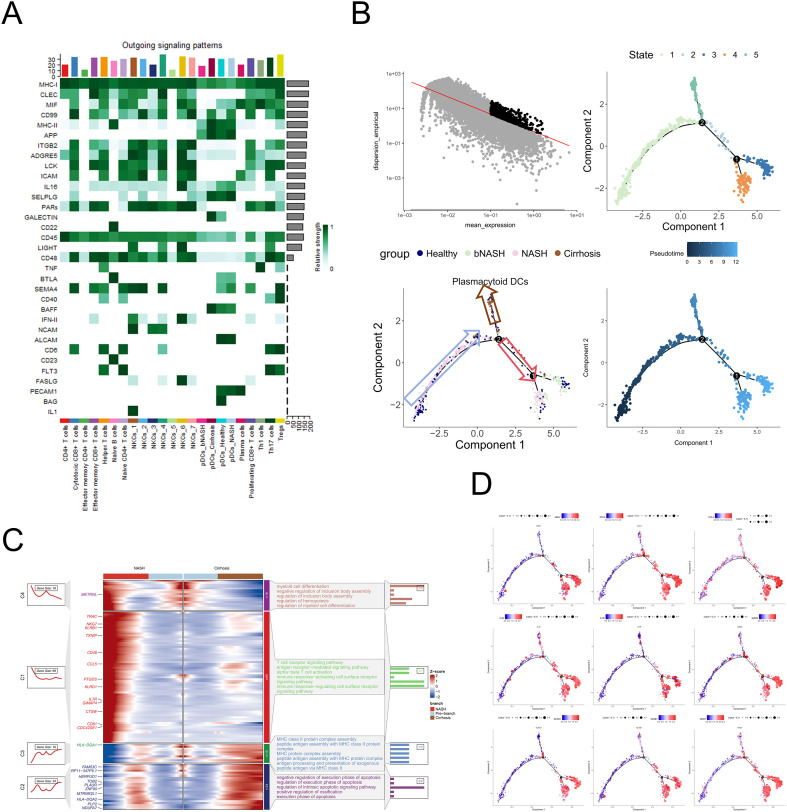
pDC lineage development in immune-driven liver pathology. **(A)** Cell–cell interaction network analysis of pDCs. **(B)** Top left, gene expression stability of pDCs; top right, pseudotime-based differentiation nodes of pDCs; bottom left, distribution of NAFLD-derived pDCs along the pseudotime axis; bottom right, inferred pseudotime developmental trajectory. **(C)** Heatmap illustrating the dynamic expression patterns of differentially expressed genes in pDCs with distinct cell fates at Branch Point 2. Rows correspond to genes; columns represent individual cells. Warmer (red) colors indicate higher gene expression, while cooler (blue) colors indicate lower expression. **(D)** Pseudo-temporal trajectory of pDC DEG dynamics, with each dot representing a single cell. Expression intensity is visualized via a gradient: red hues denote elevated gene expression. NAFLD, non-alcoholic fatty liver disease; pDC, plasmacytoid dendritic cell; DEG, differentially expressed gene.

Beyond canonical type I interferon production, pDCs exhibited robust antigen-presenting capacity and engaged in functional crosstalk with Tregs and NK cells via the BTLA and ALCAM signaling axes in NASH livers (**Figure 4A**; **Supplementary Figures 2A–F**). This signaling network likely drives immune dysregulation in NASH by amplifying inflammatory responses and fibrogenesis (**Supplementary Figures 2G, H**). These findings suggest that pDCs may modulate NASH by orchestrating crosstalk with Tregs and NKCs, a mechanism analogous to the well-documented roles of DCs in NAFLD pathogenesis (40–42). The BTLA and ALCAM signaling pathway-mediated interactions likely influence immune tolerance and cytotoxic responses, potentially amplifying inflammatory processes and fibrogenesis in the diseased liver. This functional network highlights pDCs as critical regulators of both innate and adaptive immune responses in NAFLD, offering novel insights into disease mechanisms and therapeutic targets.

### Differentiation trajectories of pDCs during NAFLD

Pseudotime trajectory analysis of myeloid lineages uncovered two critical branch points (Branch Point 1 and Branch Point 2), validated through integration with NASH-specific DEGs (**Figure 4B**). pDCs initiated divergent differentiation trajectories at Branch Point 2, with pronounced lineage segregation evident in fibrosis- and NASH-stage cells. Notably, bNASH-derived pDCs frequently clustered with NASH-stage counterparts, supporting bNASH as a transitional state. The molecular dissection of Branch Point 2-associated DEGs revealed enrichment in T-cell receptor signaling pathways (**Figure 4C**), corroborating intercellular communication findings. Longitudinal expression dynamics confirmed that genes highly upregulated along pseudotime were specifically enriched in NASH-stage pDCs (**Figure 4D**), establishing a spatiotemporal link between transcriptional programs and disease severity. These results define pDC differentiation as a continuum of functional adaptation during NAFLD progression, with Branch Point 2 marking a decisive immunometabolic transition.

### Machine learning-based diagnostic model construction using pDCs for NASH

**Figure 5:**
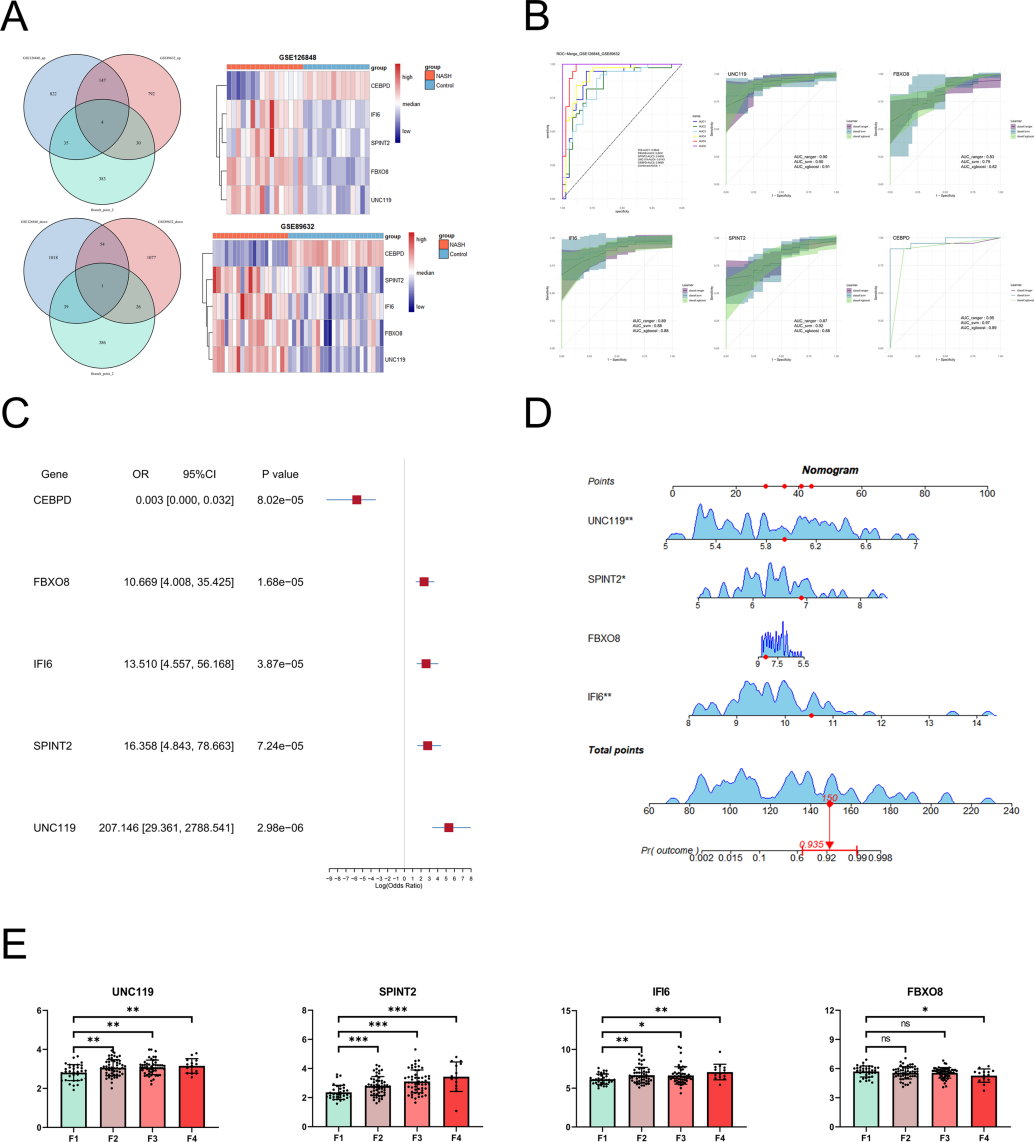
Diagnostic model for NAFLD disease progression. **(A)** Venn diagram illustrating the overlap of significantly differentially expressed genes between pDCs at Branch Point 2 and two independent GEO datasets. Four genes are commonly upregulated, and one gene is downregulated across all three datasets (left panel). Heatmap of target gene expression (CEBPD, IFI6, SPINT2, FBXO8, and UNC119) in the two GEO datasets (right panel). **(B)** ROC curves evaluating the diagnostic performance of five selected genes. **(C)** Nomogram integrating five predictive genes for estimating the probability of NASH development. **(D)** Forest plot summarizing the hazard ratios of the four genes in relation to disease progression. **(E)** Bar graph of target gene expression in NAFLD. Data derived from NASH dataset GSE135251. NAFLD, non-alcoholic fatty liver disease; pDCs, plasmacytoid dendritic cells; NASH, non-alcoholic steatohepatitis; ROC, receiver operating characteristic. *p < 0.05, **p < 0.01, ***p < 0.001.

Integrating bulk RNA-seq cohorts (GSE126848 and GSE89632), we intersected DEGs with BP2-associated pDC genes to identify five robust biomarkers: upregulated FBXO8, IFI6, SPINT2, and UNC119 and downregulated CEBPD (**Figure 5A**). Random forest, SVM, and XGBoost models achieved high diagnostic accuracy across training and validation sets (**Figure 5B**). Logistic regression confirmed inverse correlation for CEBPD and positive associations for the remaining genes (**Figure 5C**); nomogram analysis further validated UNC119, SPINT2, and IFI6 as independent NASH risk predictors (**Figure 5D**).

**Figure 6:**
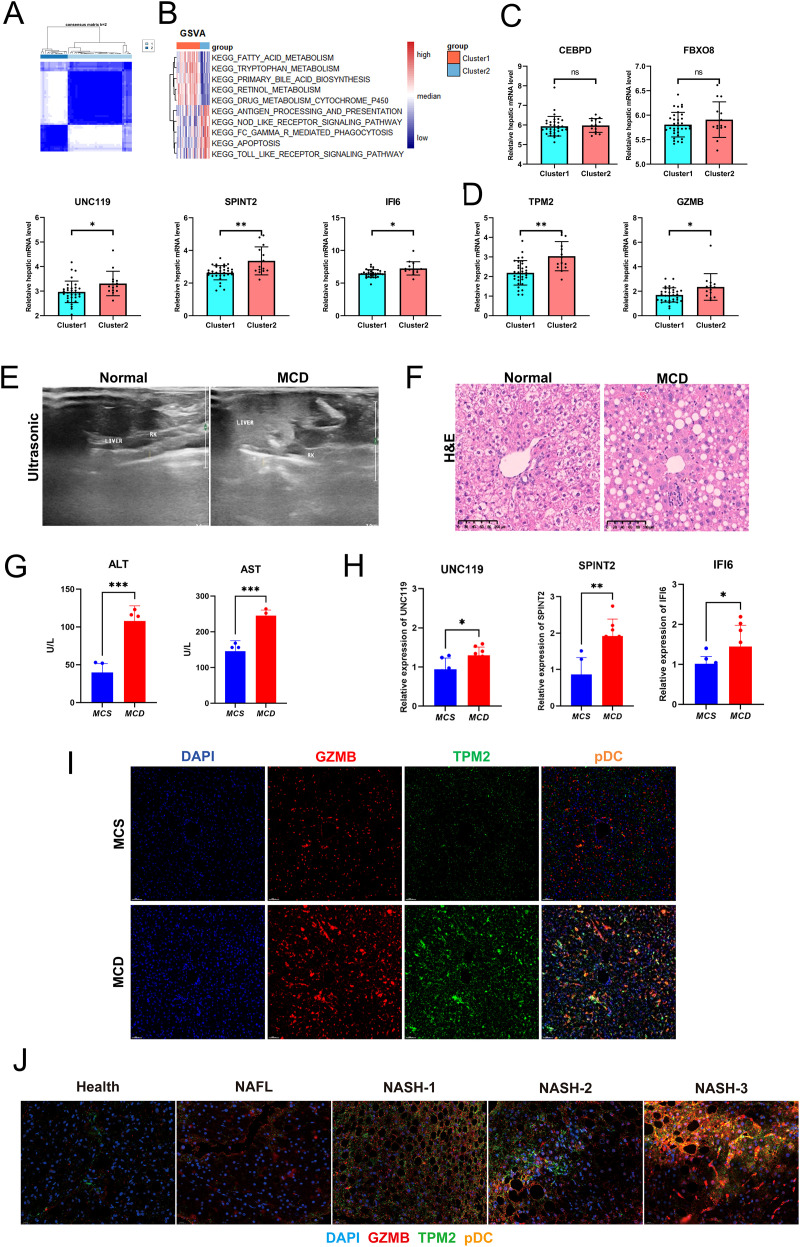
Increased GZMB^+^TPM2^+^ pDCs in human and murine NASH livers. **(A)** Unsupervised clustering analysis illustrating the distribution of cell populations across different samples. **(B)** GSVA of two clusters identified through unsupervised clustering, highlighting functional enrichment differences between these clusters. **(C)** Expression levels of key transcription factors CEBPD and FBXO8 in the two clusters. **(D)** Expression levels of UNC119, SPINT2, IFI6, TPM2, and GZMB in the two clusters. **(E)** Liver ultrasound results from mice showing structural changes in the liver between model and control groups. **(F)** H&E staining of mouse liver tissues demonstrating histopathological differences between NASH model and control groups, including steatosis and inflammation. **(G)** Serum levels of ALT and AST in mice. **(H)** Expression levels of UNC119, SPINT2, and IFI6 in mouse liver tissues. **(I)** Immunofluorescence images of GZMB^+^TPM2^+^ in mouse liver tissues. **(J)** Proportions of NASH-specific pDCs in healthy individuals and NAFLD patients. NAFLD, non-alcoholic fatty liver disease; pDCs, plasmacytoid dendritic cells; NASH, non-alcoholic steatohepatitis. *p < 0.05, **p < 0.01, ***p < 0.001.

Validation in an independent dataset (GSE135251) demonstrated sustained significance of these three genes across the F1–F4 stages (**Figure 5E**; **Supplementary Figures 3A, B**). Unsupervised clustering of the GSE167523 cohort (NASH vs. NAFL) yielded metabolism-dominant (Cluster 1) and inflammation-dominant (Cluster 2) subgroups (**Figures 6A, B**). Candidate genes and canonical pDC markers (TPM2 and GZMB) showed significant differential expression between clusters (**Figures 6C, D**). Critically, qPCR in MCD diet-induced murine NASH confirmed elevated UNC119, SPINT2, and IFI6 (**Figures 6E–H**), paralleled by increased hepatic GZMB^+^TPM2^+^ pDCs (**Figure 6I**). Immunofluorescence of human NAFLD biopsies revealed spatial co-localization of GZMB^+^TPM2^+^ pDCs within inflammatory foci and fibrotic zones (**Figure 6J**, **Supplementary Figure 3C**).

Collectively, this multi-tiered validation, spanning computational modeling, murine experimentation, and human tissue analysis, establishes GZMB^+^TPM2^+^ pDCs as a conserved NASH-specific immune signature. The integration of pseudotime-informed biomarkers with cross-platform verification provides a robust diagnostic framework and highlights pDC-targeted strategies for non-invasive NAFLD staging and immunometabolic intervention.

## Discussion

The progression from NAFL to NASH and subsequent fibrosis represents a pivotal challenge in NAFLD research (43), with dynamic immune cell remodeling serving as a central driver of this pathogenic continuum (44). Although prior studies have established immune involvement in NAFLD, stage-specific functional roles, particularly the interplay between cellular abundance and transcriptional heterogeneity, remain incompletely defined.

Integrating over >150,000 single-cell RNA sequencing profiles from the GEO database, we uncovered extensive immune landscape remodeling across the NAFLD spectrum. MCs, cDCs, and T cells exhibited stage-dependent quantitative shifts, while lineage-restricted subpopulations followed distinct evolutionary trajectories. These data position immune reprogramming not as a secondary epiphenomenon but as a mechanistic driver of liver immune microenvironment evolution. Stage-resolved transcriptional heterogeneity further offers candidate biomarkers predictive of adverse outcomes, including cirrhosis and hepatocellular carcinoma.

A central finding of this work is the identification of pDCs as critical regulators of NASH pathogenesis. Functioning as the key to antigen presentation and T-cell immunity, pDCs undergo concurrent numerical expansion and functional diversification during disease progression. Pseudotime trajectory analysis integrated with NASH-specific transcriptomic signatures identified five discriminative genes (CEBPD, FBXO8, IFI6, SPINT2, and UNC119) distinguishing NASH from NAFL. Among these, UNC119, SPINT2, and IFI6 demonstrated robust diagnostic performance, highlighting translational potential as non-invasive biomarkers.

The functional duality of dendritic cell subsets in NAFLD pathogenesis remains a subject of active investigation (45). Although cDC1 and cDC2 accumulate in NASH livers, with CXCR1^+^ cDC1 implicated in inflammatory programming, paradoxical protective roles have also been documented: Batf3-deficient mice lacking cDC1 development exhibit accelerated progression to NASH (13, 46), whereas adoptive transfer of CD103^+^ cDC1 ameliorates this phenotype (42). This context-dependent functionality underscores the complexity of conventional DC biology in NAFLD. In stark contrast, the contribution of pDCs remained entirely uncharacterized (41, 47). To address this gap, we employed an MCD diet-induced NAFLD mouse model and utilized GZMB^+^/TMP2^+^ surface markers to precisely localize hepatic pDCs. Their specific enrichment within peri-vascular niches, coupled with a significant positive correlation between cellular abundance and histological inflammation scores, provides compelling spatial evidence positioning pDCs as critical mediators of immune-driven hepatic injury.

Integrating these spatial insights with deep transcriptional profiling across public single-cell datasets, we delineated profound numerical expansion and functional reprogramming of pDCs throughout NAFLD progression. Pseudotime trajectory analysis corroborated the five signature genes previously identified, with UNC119, SPINT2, and IFI6 exhibiting robust diagnostic accuracy for NASH. These findings solidify pDCs as pivotal orchestrators of immune microenvironment remodeling and validate their translational promise as non-invasive biomarkers.

Collectively, we propose a refined immunopathogenic framework for NAFLD in which dynamic pDC expansion and differentiation constitute both a disease-stage hallmark and a therapeutically tractable node. The pDC-associated gene signatures (UNC119, SPINT2, and IFI6) provide a molecular foundation for non-invasive NASH detection and precise staging, effectively bridging computational inference with experimental validations across murine models and human liver biopsies. Future investigations should delineate mechanistic crosstalk between pDCs and other immune subsets, particularly Tregs and NK cells, while assessing translational utility in clinical diagnostics. Crucially, stage-resolved validation of BTLA and ALCAM expression dynamics on liver-infiltrating pDCs, across NAFLD stages, combined with functional interrogation in conditional knockout models, will be essential to indispensable for establishing causal relationships and assessing checkpoint modulatory strategies for NASH intervention.

## Data Availability

Publicly available datasets were analyzed in this study. This data can be found here: GSE136103 (https://www.ncbi.nlm.nih.gov/geo/query/acc.cgi?acc=GSE136103), GSE115469 (https://www.ncbi.nlm.nih.gov/geo/query/acc.cgi?acc=GSE115469), GSE159977 (https://www.ncbi.nlm.nih.gov/geo/query/acc.cgi?acc=GSE159977), GSE126848 (https://www.ncbi.nlm.nih.gov/geo/query/acc.cgi?acc=GSE126848), GSE89632 (https://www.ncbi.nlm.nih.gov/geo/query/acc.cgi?acc=GSE89632), GSE135251 (https://www.ncbi.nlm.nih.gov/geo/query/acc.cgi?acc=GSE135251), GSE167523 (https://www.ncbi.nlm.nih.gov/geo/query/acc.cgi?acc=GSE167523).

## Supplementary material

Supplementary Figure 1 **(A)**, UMAP plots of different myeloid immune cell markers, **(B)**, UMAP plots of different lymphoid immune cell markers, **(C)**, KEGG enrichment Venn diagram of pDCs at different stages of NAFLD, **(D)**, Bubble plot showing BP enrichment results for NASH-specific pDCs.

Supplementary Figure 2 **(A)**, Cell communication network between NASH-specific pDCs and myeloid immune cells, **(B)**, Representative BTLA signaling interaction pattern among myeloid immune cells, **(C)**, Expression levels of BTLA signaling-related proteins TNFRSF14 and BTLA in myeloid cells and pDCs, further supporting their roles in immune regulation. **(D)**, Cell communication network between NASH-specific pDCs and lymphocytes, **(E)**, Representative ALCAM signaling interaction pattern among lymphocytes, demonstrating the regulatory role of ALCAM signaling in lymphocytes. **(F)**, Expression levels of ALCAM signaling-related proteins CD6 and ALCAM in lymphocytes and pDCs, **(G)**, Heatmap of signaling pathway patterns in each cluster of cells, **(H)**, Expression levels of pDC-related genes based on pseudotime analysis.

Supplementary Figure 3 **(A, B)**, Expression levels of TPM2 and GZMB in different stages of liver fibrosis, **(C)**, Levels of pDCs in healthy individuals and NAFLD patients, comparing the differences in pDC numbers and their clinical significance between the two groups.
