# Supplementary figures and images for: Dynamic immune landscape of NAFLD highlights pDCs as predictive biomarkers for disease progression
Source: Front Immunol. 2026 Apr 10;17:1702007. doi: 10.3389/fimmu.2026.1702007 (PMC13105974; doi:10.3389/fimmu.2026.1702007)

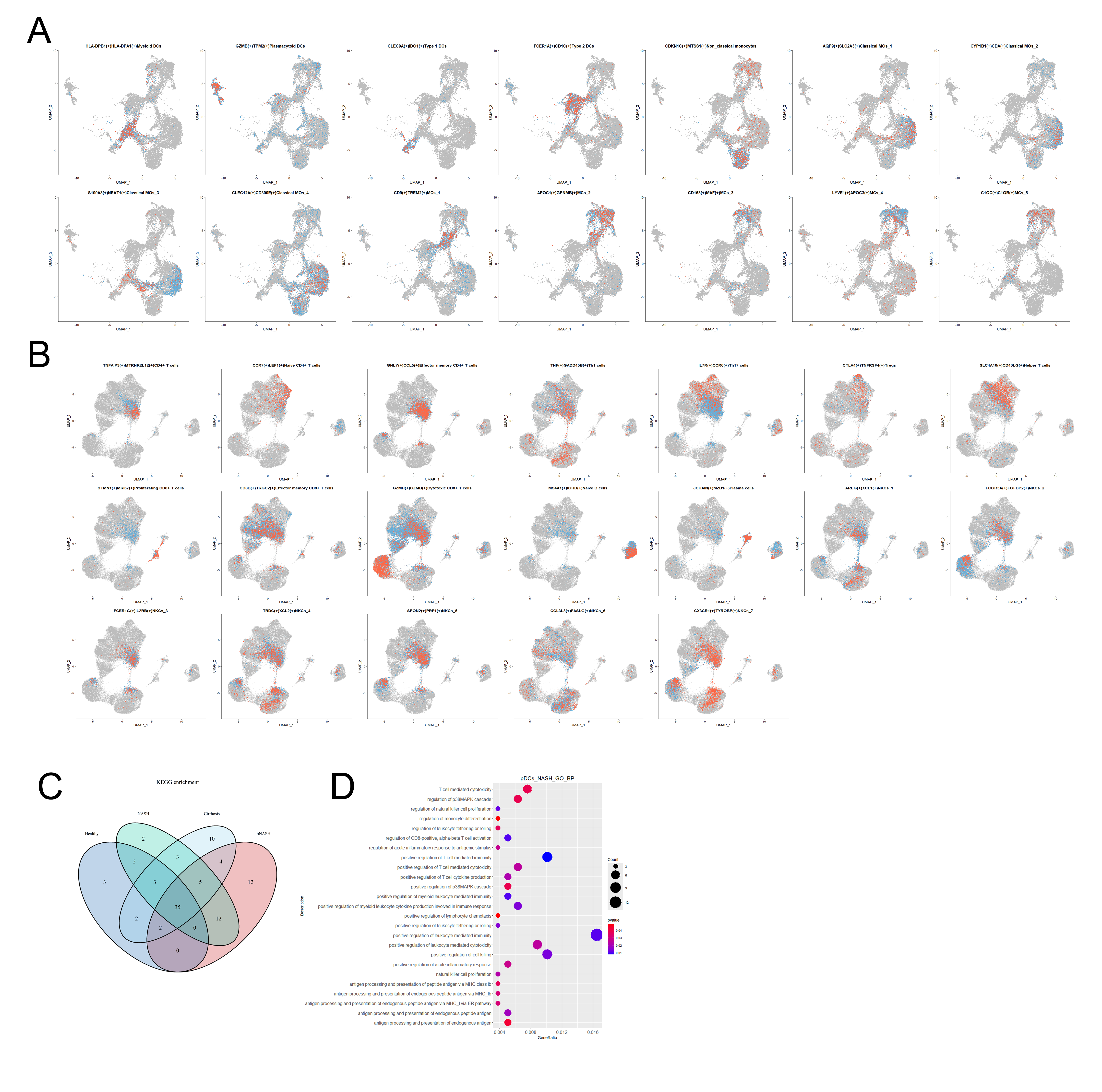

Supplement: Supplementary Figure 1 — (A), UMAP plots of different myeloid immune cell markers, (B), UMAP plots of different lymphoid immune cell markers, (C), KEGG enrichment Venn diagram of pDCs at different stages of NAFLD, (D), Bubble plot showing BP enrichment results for NASH-specific pDCs. [file Image1.tif]

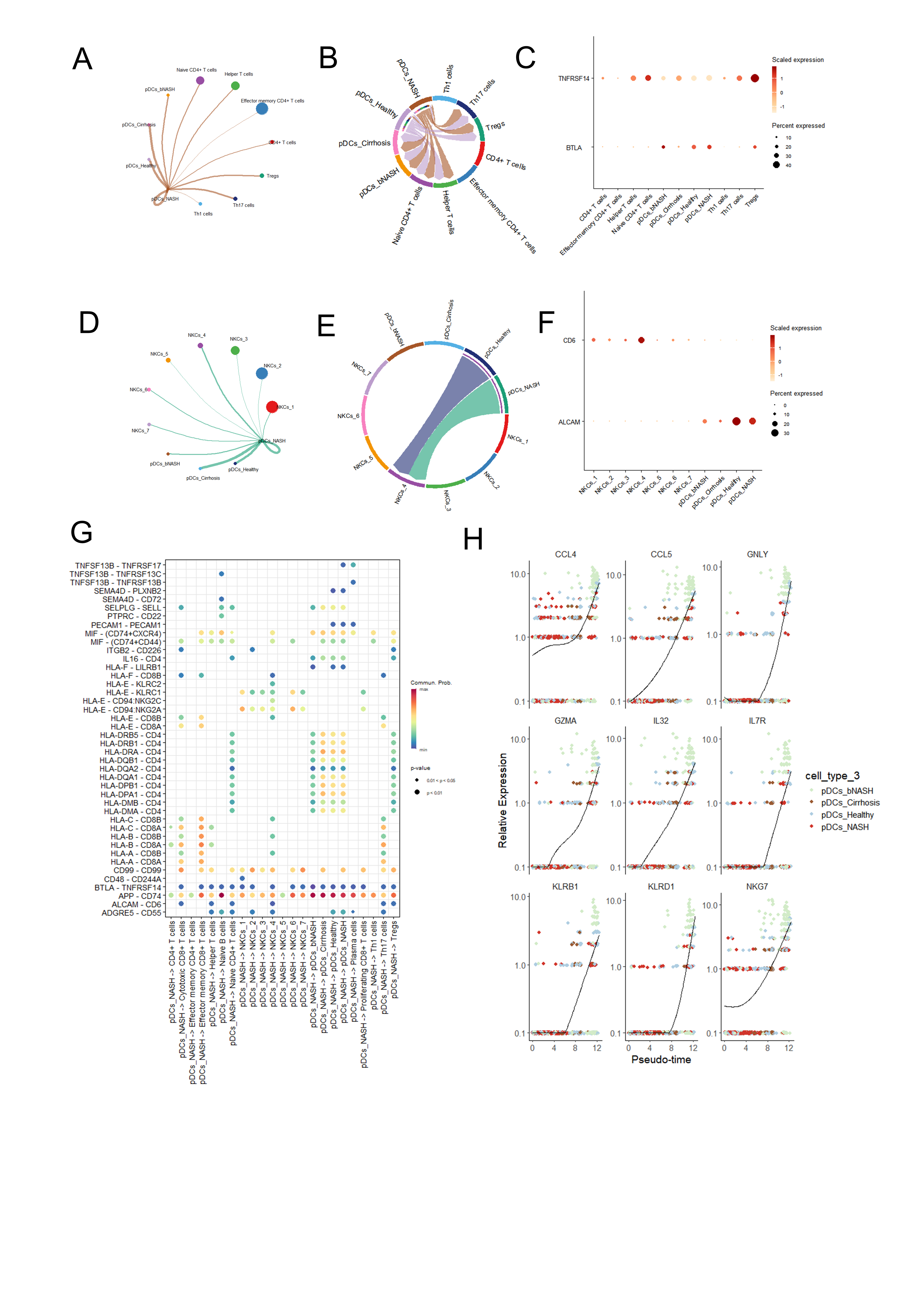

Supplement: Supplementary Figure 2 — (A), Cell communication network between NASH-specific pDCs and myeloid immune cells, (B), Representative BTLA signaling interaction pattern among myeloid immune cells, (C), Expression levels of BTLA signaling-related proteins TNFRSF14 and BTLA in myeloid cells and pDCs, further supporting their roles in immune regulation. (D), Cell communication network between NASH-specific pDCs and lymphocytes, (E), Representative ALCAM signaling interaction pattern among lymphocytes, demonstrating the regulatory role of ALCAM signaling in lymphocytes. (F), Expression levels of ALCAM signaling-related proteins CD6 and ALCAM in lymphocytes and pDCs, (G), Heatmap of signaling pathway patterns in each cluster of cells, (H), Expression levels of pDC-related genes based on pseudotime analysis. [file Image2.tif]

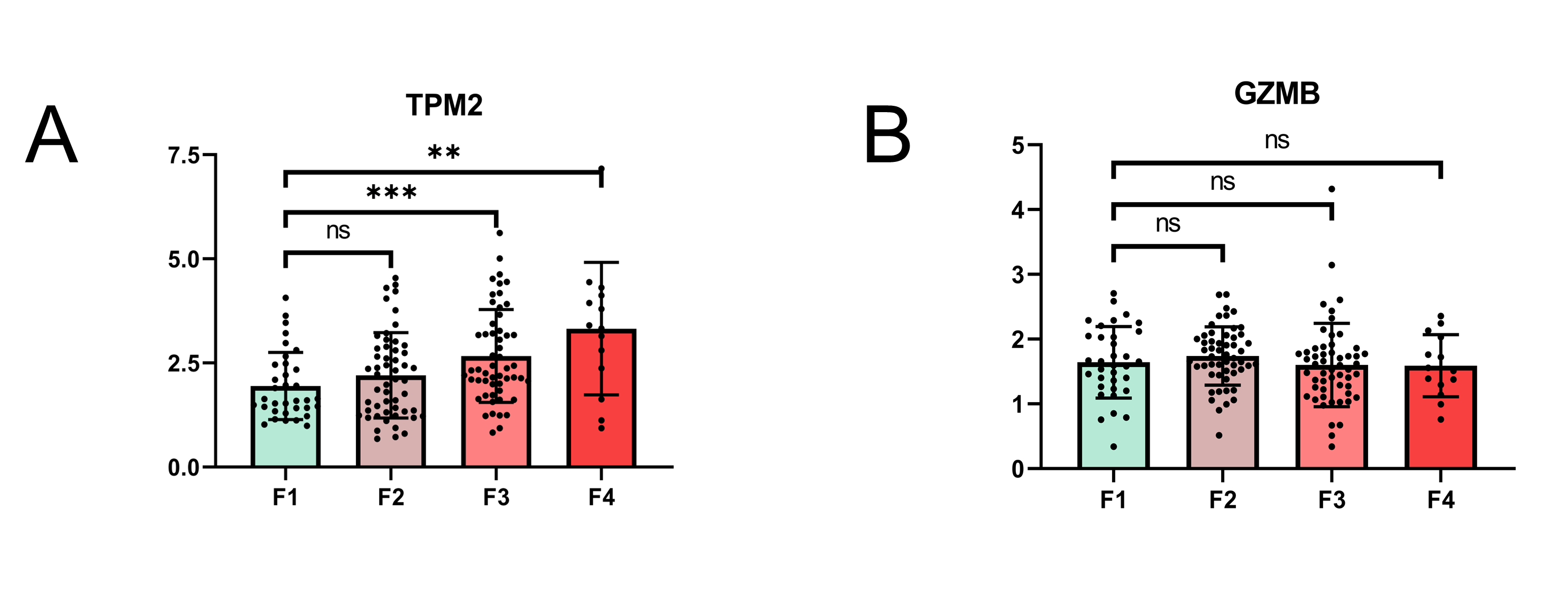

Supplement: Supplementary Figure 3 — (A, B), Expression levels of TPM2 and GZMB in different stages of liver fibrosis, (C), Levels of pDCs in healthy individuals and NAFLD patients, comparing the differences in pDC numbers and their clinical significance between the two groups. [file Image3.tif]
